# Analysis of *ACE2* genetic variants in 131 Italian SARS-CoV-2-positive patients

**DOI:** 10.1186/s40246-020-00279-z

**Published:** 2020-09-11

**Authors:** Antonio Novelli, Michela Biancolella, Paola Borgiani, Dario Cocciadiferro, Vito Luigi Colona, Maria Rosaria D’Apice, Paola Rogliani, Salvatore Zaffina, Francesca Leonardis, Andrea Campana, Massimiliano Raponi, Massimo Andreoni, Sandro Grelli, Giuseppe Novelli

**Affiliations:** 1 Laboratory of Medical Genetics, Bambino Gesù Children’s Hospital, IRCCS, Rome, Italy; 2 Department of Biology, Tor Vergata University of Rome, 00133 Rome, Italy; 3 Medical Genetics Laboratory, Tor Vergata Hospital, Rome, Italy; 4 Department of Biomedicine and Prevention, Tor Vergata University of Rome, 00133 Rome, Italy; 5 Unit of Respiratory Medicine, Department of Experimental Medicine, University of Rome “Tor Vergata”, Rome, Italy; 6 Occupational Medicine, Bambino Gesù Children’s Hospital, IRCCS, Rome, Italy; 7 Intensive Care Unit, Tor Vergata University Hospital, Rome, Italy; 8 Department of Pediatrics, IRCCS “Bambino Gesù” Children’s Hospital, Rome, Italy; 9 Helth Directorate Bambino Gesù Children’s Hospital, IRCCS, Rome, Italy; 10 Department of Systems Medicine, University of Rome Tor Vergata, Rome, Italy; 11 Infectious Diseases Clinic, Policlinico Tor Vergata, Rome, Italy; 12 Department of Experimental Medicine and Biochemical Sciences, University of Rome Tor Vergata, Rome, Italy; 13 IRCCS Neuromed, Pozzilli (IS), Italy; 14 Department of Pharmacology, School of Medicine, University of Nevada, Reno, NV 89557 USA

## Abstract

**Background:**

Coronaviruses (CoV) are a large family of viruses that are common in humans and many animal species. Animal coronaviruses rarely infect humans with the exceptions of the Middle East respiratory syndrome (MERS-CoV), the severe acute respiratory syndrome corona virus (SARS-CoV), and now SARS-CoV-2, which is the cause of the ongoing pandemic of coronavirus disease 2019 (COVID-19). Several studies suggested that genetic variants in the *ACE2* gene may influence the host susceptibility or resistance to SARS-CoV-2 infection according to the functional role of ACE2 in human pathophysiology. However, many of these studies have been conducted in silico based on epidemiological and population data. We therefore investigated the occurrence of *ACE2* variants in a cohort of 131 Italian unrelated individuals clinically diagnosed with COVID-19 and in an Italian control population, to evaluate a possible allelic association with COVID-19, by direct DNA analysis.

**Methods:**

As a pilot study, we analyzed, by whole-exome sequencing, genetic variants of *ACE2* gene in 131 DNA samples of COVID-19 patients hospitalized at Tor Vergata University Hospital and at Bambino Gesù Children’s Hospital, Rome. We used a large control group consisting of 1000 individuals (500 males and 500 females).

**Results:**

We identified three different germline variants: one intronic c.439+4G>A and two missense c.1888G>C p.(Asp630His) and c.2158A>G p.(Asn720Asp) in a total of 131 patients with a similar frequency in male and female. Thus far, only the c.1888G>C p.(Asp630His) variant shows a statistically different frequency compared to the ethnically matched populations. Therefore, further studies are needed in larger cohorts, since it was found only in one heterozygous COVID-19 patient.

**Conclusions:**

Our results suggest that there is no strong evidence, in our cohort, of consistent association of *ACE2* variants with COVID-19 severity. We might speculate that rare susceptibility/resistant alleles could be located in the non-coding regions of the *ACE2* gene, known to play a role in regulation of the gene activity.

## Introduction

Since the end of last year, in December 2019, Chinese authorities have reported several cases of pneumonia in Wuhan City, Hubei Province of China [1]. A novel betacoronavirus was identified as the causative agent of the viral acute respiratory human distress [2, 3]. Afterwards, the disease was named “Coronavirus Disease 2019 (COVID-19)” by the World Health Organization (WHO) [4].

Coronaviruses (CoV) are a large family of viruses that are common in humans and other animal species, including bats [5], camels, cattle, and cats. Animal coronaviruses rarely infect humans and then spread between subjects with the exceptions of the Middle East respiratory syndrome (MERS-CoV), the severe acute respiratory syndrome coronavirus (SARS-CoV), and now SARS-CoV-2, which is the cause of the ongoing pandemic [6]. A critical step for a viral infection is receptor recognition and binding to the host-cell surface. The angiotensin-converting enzyme 2 (ACE2) has been identified as a functional receptor for SARS-CoV-2, allowing host-cell entry [7]. SARS-CoV-2 uses an extensively glycosylated spike (S) protein that protrudes from the viral envelope and mediates the binding to ACE2 [5], the carboxypeptidase that catalyzes the hydrolysis of angiotensin II to angiotensin (1-7) [8]. The S protein is a 1273 amino acid (aa) long-structural glycoprotein located on the outer envelope of the virus. It has two functional subunits: an N-terminal S1 subunit and a shorter C-terminal S2 subunit. ACE2 is a single-pass type I membrane protein (805 aa) and it contains an N-terminal peptidase M2 domain and a C-terminal collectrin domain. The binding affinity of the ACE2 receptor-binding domain (RBD) to the C-terminal domain of S1 subunit of the SARS-CoV-2 S protein is 10- to 20-fold higher than that of SARS-CoV, which may contribute to the higher infectivity and transmissibility of SARS-CoV-2 [9–11]. The high variation in clinical severity observed among patients may be suggestive of a critical role of the inter-individual variability in the host genetic background. It is consequently conceivable that the distribution of ACE2 determines the virus cell and tissue tropism and pathogenesis [12]. Investigating the molecular virus-receptor interactions is a crucial step through the understanding of viral pathogenesis and host susceptibility. Clinical conditions include pulmonary and extrapulmonary manifestations. Despite a standardized molecular mechanism of infection, SARS-CoV-2 shows highly variable clinical presentations, amenable to a variety of factors that range from viral strain to the host genetic background [12]. Indeed, several studies inferred that genetic variants in *ACE2* gene may influence the individual susceptibility or resistance to SARS-CoV-2 according to the functional role of ACE2 in human pathophysiology [12]. It is possible that the affinity of binding of SARS-CoV-2 to ACE2 could be modulated by genetic variants within the RBD and/or other ACE2 domains. As a matter of fact, genetic variants affecting the receptor might influence infection rates and severity of the disease. *ACE2* functional variants might enhance or reduce the binding affinity for the RBD by altering the residues accessibility or protein conformation. Seeking for variants that show a correlation with the disease severity and investigating their influence on the viral replicative cycle is a first step to unveil the reasons behind the broad range of disease outcomes. Furthermore, it might provide insights for the development of antiviral therapies.

In this study, we, therefore, investigated the occurrence of *ACE2* variants in a cohort of 131 Italian SARS-CoV-2-positive patients, extracting data on *ACE2* variants by direct DNA analysis. We also verified the existence of an association of *ACE2* variants with severity of the disease.

## Materials and methods

### Clinical study

For our study, we enrolled a total of 131 subjects with COVID-19. More than half were hospitalized at Tor Vergata University Hospital (*n* = 89, 68%) and the remaining at Bambino Gesù Children’s Hospital of Rome (*n* = 42, 32%). One hundred fourteen patients (87%) showed clinical symptoms of COVID-19. All were diagnosed with COVID-19 after positive results of naso-oropharyngeal swabs. They were admitted to the relevant wards for appropriate care and checks, while the asymptomatic subjects (*n* = 17, 13%) returned home or were kept under brief observation for few days.

Most of the enrolled subjects were male (81/131; 62%). Age ranged between 6 and 92 years old (median age ± SD 57 ± 19.7). Fifty subjects were female (38%), with their age ranging from 2 to 93 years old (median age ± SD 55 ± 22.9). 13 were children (median age ± SD 11 ± 4.2), classified as asymptomatic or with mild disease severity. None of the patients showed symptoms of Kawasaki-like syndrome [13].

We clustered all the patients in four disease severity group as stated by their hospitalization outcomes:

- *Asymptomatic*: absence of clinical symptoms (*n* = 17, 13%; median age ± SD 39 ± 16.2 years old);
- *Mild*: presence of few symptoms, but not requiring ventilation, except for cases of respiratory support via Venturi Mask (VMK) (*n* = 16, 12%; median age ± SD 48 ± 23.3 years old);
- *Moderate*: showing respiratory impairment, requiring non-invasive ventilation and CPAP (continuous positive airway pressure) or BiPAP (bilevel positive airway pressure) cycles (*n* = 43, 33%; median age ± SD 61 ± 14.7 years old);
- *Severe*: defined as respiratory failure, requiring invasive ventilation and intensive care unit (ICU) admission (*n* = 55, 42%; median age ± SD 65 ± 17.6 years old).

Venous blood samples from patients and control individuals (1000 Italian subjects, 500 males, and 500 females) were collected for the Whole Exome Sequencing (WES).

Our investigations received approval by the local ethics committee at Tor Vergata University Hospital (protocol no. 50/20). The study was conducted in agreement with the principles of the Declaration of Helsinki. Informed written consent was obtained from each patient.

### Whole exome sequencing and data preprocessing

Library preparation and whole exome capture were performed by using the Twist Human Core Exome Kit (Twist Bioscience) according to the manufacture's protocol and sequenced on the Illumina NovaSeq 6000 platform. The BaseSpace pipeline (Illumina) and the TGex software (LifeMap Sciences) were used for the variant calling and annotating variants, respectively. Sequencing data were aligned to the hg19 human reference genome. Based on the guidelines of the American College of Medical Genetics and Genomics (ACMG), a minimum depth coverage of 30X was considered suitable for analysis. Variants were examined for coverage and Qscore (minimum threshold of 30), and visualized by the Integrative Genome Viewer (IGV). For this study, we analyzed only data on the *ACE2* candidate gene.

### Statistical analysis

Differences in alleles frequencies between groups were evaluated by the Pearson *χ2* test or by Fisher’s exact test, as requested according to the numbers of samples in the compared groups. *P* values less than 0.05 were considered statistically significant. Since we considered only *ACE2* gene, with a “candidate gene” approach, we did not perform corrections for multiple comparison normally used for exome sequencing data analyses of thousands of genes. The Hardy-Weinberg equilibrium was evaluated, where possible, by the Pearson *χ2* test.

## Results

We identified three different germline variants, one intronic c.439+4G>A (rs2285666) and two missense c.1888G>C p.(Asp630His) (rs140312271) and c.2158A>G p.(Asn720Asp) (rs41303171), in a total of 30 patients (14 females and 16 males). Seven out of 30 were asymptomatic (23%; median age ± SD 42 ± 19.4 years old); 3 out of 30 were mild (10%; median age ± SD 15 ± 32.7 years old); 6 out of 30 were moderate (20%; median age ± SD 66 ± 19.1 years old); and 14 out of 30 were severe (median age ± SD 70.5 ± 10.6 years old). Four out of 30 passed away (1 male and 3 female; median age ± SD 74 ± 11.9 years old). The frequency of the three identified variants are similar between male and female patients suggesting also there is no gender effect underlying the frequency distribution of *ACE2* variants (Table 1). GnomAD database analysis revealed that these identified *ACE2* variants are reported with a cumulative frequency of 0.2289 in ethnically matched populations (EUR). The cumulative frequency of these variants in our examined Italian cohort is 0.2353 and is not statistically different (Table 1). A significant difference was detected only for the c.1888G>C p.(Asp630His) even if this result is to be confirmed in a larger cohort since it was found only in a heterozygous female (*p* = 0.0088) (Table 1). The allelic frequency of this variant in GnomAD for the EUR reference population is 0.0000368 confirming that this is a very rare allele. This variant was not found in our Italian control population. In order to predict the functional impact of this variant on the protein, we used several tools (PolyPhen2, Mutation Taster, SIFT) and two ensemble score (MetaLR_pred, MetaSVM_pred.). The in silico analysis gave conflicting computational verdicts because of 3 benign predictions vs. 2 pathogenic predictions. The sequence alignment of the ACE2 protein with its orthologous proteins shows that the wild type residue is not highly conserved in species implying an irrelevant functional or structural role of this residue in the ACE2 protein. However, this variant deserves further investigation in a larger COVID-19 cohorts as well as functional studies. Concerning the other two variants, the recurrent c.439+4A>G (rs2285666) intronic variant has been previously reported by Strafella et al. [14] and by Asselta et al. [15] in two different Italian cohorts representative of the country’s population. The variant is located in the intron 3 in a splice site region of the gene. However, using Human Splicing Finder (HSF) no significant splicing alterations were suggested. The missense variant c.2158A>G p.(Asn720Asp) was found in two patients, one female in heterozygous state and one male, with a frequency in line with our Italian control population and with the frequency reported for the European non-Finnish population in the GnomAD database. This variant is located in the C-terminal domain, which is not involved in the SARS-CoV-2 S protein interaction. The in silico analysis to predict the potential impact of this variant on the protein sequence gave benign computational verdict because of 4 benign predictions vs. 1 pathogenic prediction. We tested the hypothesis if these variants were associated with COVID-19 severity. We analyzed the SNP rs2285666 (for which we observed more variant alleles) comparing both asymptomatic *vs* mild-moderate-severe and severe *vs* moderate-mild asymptomatic, but we did not find any kind of significant difference (data not shown). For the other two SNPs, allelic variants were observed only in asymptomatic subjects in heterozygous status. However, the small number of patients in each subgroup considered does not allow us to make definitive conclusions.

**Table 1:** Allelic counts (variants vs WT alleles) and allelic frequencies of ACE2* variants found in our Italian population of SARS-CoV-2-positive patients. The data of the same variants are reported also for the Italian control population and in Europeans (GnomAD database)

Nr.	dbSNP	Position (Hg19)	Coding	Protein	Genotype	Variant type	Allelic count	Allelic count EUR (GnomAD)	Allelic count Italian controls	Allelic frequency	Allelic frequency EUR (GnomAD)	Allelic frequency Italian controls	***p*** value COVID-19 vs EUR (GnomAD)	***p*** value COVID-19 vs Italian controls
					(***n***)									
1	**rs140312271**	chrX:15588426	c.1888G>C	p.(Asp630His)	Het (1)	Missense variant	1	3		1/181	3/81442		*p* = **0.0088**	/
							vs	vs	*Not found*	= 0.0055	= 3.68x10^-5^	*NOT FOUND*		
							180	81439						
2	**rs2285666**	chrX:15610348	c.439+4G>A		Hom (4)	Splice region variant	32	17240	338	32/181	17240/86164	338/1500		
					Het (9)		vs	vs	vs	= 0.1768	= 0.2001	= 0.2253	*p* = 0.43	*p* = 0.14
					Hem (15)		149	68924	1162					
3	**rs41303171**	chrX:15582298	c.2158A>G	p.(Asn720Asp)	Het (1)	Missense variant	2	2195	15	2/181	2195/87066	15/1500		
					Hem (1)		vs	vs	vs	= 0.011	= 0.0251	= 0.01	*p* = 0.34	*p* = 0.70
							179	84871	1485					
**Cumulative frequencies (variants 1, 2, 3)**							35	19438	353	35/181	19438/84890	353/1500		
The allelic count of Europeans wild-type alleles (GnomAD database) in cumulative frequencies was calculated as the average of the number of wild-type alleles of the three genetic variants, since the 3 variants has been genotyped in a number of individuals slightly different							vs	vs	vs	= 0.1934	= 0.2289	= 0.2353	*p* = 0.86	*p* = 0.21
							146	78411	1147					

*The ACE2 reference sequences used are NM_021804.3 and NG_012575.1

## Discussion

Several in silico data suggested that the ACE2 variants in structural part of the protein could have an impact on the pathogen binding dynamics or increase the quantitative expression of *ACE2* [7, 10–12]. ACE2 receptor binds the SARS-CoV-2 spike protein at least 10 times more tightly than SARS-CoV-1 [16]. This might explain some of the differences between the two viruses in the way they infect people and cause disease. For this reason, we wanted to analyze in detail the genetic variability of ACE2 in our population, among the most affected by the COVID-19 pandemic. Until now, most available published studies were carried out on an epidemiological basis of population allele frequencies deposited in the various available databases. We first tested the hypothesis on the existence of an enrichment of coding-region variants in *ACE2* gene, able to affect the binding dynamics of SARS-CoV-2 to the receptor. Despite the number of patients systematically analyzed in this series, it is small for definitive conclusions, we believe that there are no enrichments of rare functional alleles in ACE2 capable of influencing the binding capacity of the receptor. We identified in a single COVID-19 patient a variant (p.Asp630His), very rare in European population and not detected in our Italian control population. Similarly, we did not observe significant differences by stratifying patients according to the clinical phenotype. In fact, 28 out of 30 patients (93%), with a asymptomatic, mild, moderate, and severe outcomes, presented c.439+4G>A variant; 2 asymptomatic out 30 patients (6%) presented c.2158A>G, p.Asn720Asp variant; only one asymptomatic woman (1%) presented c.1888G>C, p.Asp630His variant. However, these preliminary results should be verified in a much larger cohort for a more accurate risk assessment.

Our results confirm and extend the knowledge that *ACE2* is a gene with a low allelic frequency of missense variants as expected on the basis of GnomAD population data. In fact, we provide evidence that the rate of amino acid changes at the binding region with SARS-CoV-2 and at the protein cleavage sites is very low. This suggests that these regions have been under evolutionary pressure, probably for the essential catalytic role of ACE2 as transmembrane carboxypeptidase. It is possible that rare susceptibility alleles are located in the non-coding regions of the gene, involved in the regulation of *ACE2* gene activity. Also, a recent GWA study on a high number of patients did not show evidence of association with *ACE2* variability [17]. Mutant alleles in non-coding DNA can cause alterations in expression levels or timing. These variations concern enhancers, promoters, insulators, and silencers or regions that provide instructions for producing functional RNA molecules, such as transfer RNA, miRNAs, or long non-coding RNA [18]. By inspecting the human genetic variants pool available at https://www.ncbi.nlm.nih.gov/snp/, ∼ 16,493 SNPs were extracted after filtering for the non-coding regions of *ACE*2. We are aware that the totality of these variants has no functional meaning. However, some of these may influence the expression of the receptor in a tissue-dependent way. It is therefore of interest to explore the existence of *ACE2* susceptibility alleles to SARS-CoV-2 in these regulatory regions. Interestingly very recently, Bunyavanich et al. [19] showed age-dependent expression of *ACE2* gene in nasal epithelium, highlighting that the different levels of ACE2 expression may be the reason for a lower incidence of COVID-19 in children. Several studies have shown that *ACE2* gene undergoes the action of at least four miRNAs: miR-200c, let-7b, miR-1246, and miR-125b [20–23]. Polymorphisms within genes coding for these miRNAs could be of great help with regards to investigations on the regulation of *ACE2* gene expression and the possible significance of variations in further more in depth studies.

## Conclusions

Our study suggests that there is no strong evidence, in our cohort, of consistent association of *ACE2* genomic variants with COVID-19 susceptibility or clinical phenotype. However, we cannot rule out a type II error considered to be a relatively small size of the samples tested. Despite this, we might speculate that rare susceptibility alleles could be located in the non-coding regions of the *ACE2* gene, known to have a role in regulating gene activity. It should be therefore interesting to explore the existence of *ACE2* susceptibility alleles to SARS-CoV-2 in the regulatory regions of the gene.
